# Supplementary material for: Integrated Analysis of Small RNA, Transcriptome, and Degradome Sequencing Reveals the Water-Deficit and Heat Stress Response Network in Durum Wheat
Source: Int J Mol Sci. 2020 Aug 21;21(17):6017. doi: 10.3390/ijms21176017 (PMC7504575; doi:10.3390/ijms21176017)
Supplement: Supplementary file 1 [file ijms-21-06017-s001.zip › Supplementary files R2/Supplemental experimental procedures.pdf]

Supplemental experimental procedures

Small RNA sequencing and data analysis

Small RNA sequencing analysis was performed using the ACGT101-miR program (LC Sciences, USA) as previously described [1]. Raw reads were first processed by removing low-quality reads and adapter sequences to obtain clean sequences. Reads with a nucleotide (nt) length <18 nt or >25 nt were removed. Non-coding (nc) RNA families (rRNA, tRNA, snRNA and snoRNA), repeats and mRNA sequences were discarded using RFam, Repbase and durum wheat NCBI mRNA entries as references. Unique sRNA sequences were then obtained for each library. Conserved mature miRNAs and 5p- or 3p-derived miRNA variants were identified using BLAST search against the plant miRBase (Release 22.1). MIR (microRNA gene locus) and miRNA sequences from common plant species in the miRBase were used as references. Sequences mapped to the mature miRNA in the hairpin were identified as conserved mature miRNAs. Sequences mapped to the opposite arm of mature miRNA in the hairpin were identified as 5p- or 3p-derived variants. Single mismatch within the sequence and length variation at both 5p and 3p were allowed in the alignment. All the mapped miRNAs were aligned to the durum wheat genome (NCBI UID 3439611, assembly Svevo.v1) to determine their genomic location.

The remaining unmatched sRNA sequences were used to identify novel durum miRNAs. Sequences were BLASTed to the durum wheat genome. To identify miRNA precursors, secondary hairpin structures containing matched sequences were predicted using RNAfold (http://rna.tbi.univie.ac.at/cgi-bin/RNAfold.cgi) from 120nt of flanking genome sequences. The following criteria were used for the prediction of the secondary structure as previously described [1]: 1) the length of the hairpin (two stems and the terminal loop) is ≥ 50nt; 2) the length of the hairpin loop region is ≤ 200nt; 3) the cut-off of free energy is ≤ −15kCal/mol; 4) the stem region of the predicted hairpin is ≥ 16bp in length; 5) for each bulge in the stem, the nucleotide length is ≤ 12nt; 6) for each bulge in the mature region of the predicted hairpin, the nucleotide length is ≤ 4nt; 7) the number of biased matches in each bulge in the mature region is ≤ 2; (8) the number of biased bulges in the mature region is ≤ 2; 9) the number of mismatches in the mature region is ≤ 4; 10) the mature region is ≥ 12bp in length; and 11) the mature region in the stem is ≥ 80%.

All miRNAs were categorised into five groups (G1-5) [1]. The criteria of categorisation are as follows: G1, reads-aligned pre-miRNAs can be mapped to the durum genome and ESTs (expressed sequence tags). G2, sequencing reads can be mapped to the durum genome and ESTs, with their extended genome sequences forming secondary hairpins. G3, sequencing reads can be mapped to the durum genome and ESTs, but their extended genome sequences do not form secondary hairpins. G4, neither aligned reference pre-miRNAs nor sequencing reads can be further mapped to the genome. G5, novel miRNAs where reads cannot be mapped to the miRBase but can be mapped to the genome, and secondary hairpins can be formed from extended genome locations. To analyse the miRNA expression profiles, data normalisation of the reads number was carried out as previously described [2].

Transcriptome sequencing and data analysis

Prior to sequence assembly, low quality reads (those containing primer or adaptor sequence, and those with a sequencing quality score < 20) were removed. Clean reads were aligned to the durum reference genome using the HISAT package (V2.0). Aligned reads of each library were assembled and the transcript abundance were obtained using StringTie (V1.3.0). Normalised relative abundance was expressed in FPKM (Fragments Per Kilobase Million).

Degradome sequencing and data analysis

Approximately 20 μg of total RNA per sample was used for library construction. First, mRNA was enriched using oligo-d(T) magnetic beads. The enriched mRNA was mixed with biotinylated

random primers and were ligated to 5' adaptors. First-strand cDNA was reverse-transcribed from ligated mRNA products and then amplified with PCR. The final cDNA libraries were sequenced on an Illumina Hiseq2500 (LC-BIO, Hangzhou, China). Raw sequencing reads were processed using the ACGT101-DEG program (V4.1, LC Sciences, USA) to remove low-quality reads, reads with adaptor and primer contamination, and reads that can be annotated as non-coding RNA families. The remaining clean reads were used to identify the degraded fragments of mRNAs that are targets of known and novel durum miRNAs with the CleaveLand package V4.0 (Addo-Quaye et al., 2009a,b) and the ACGT101-DEG program (LC Sciences, TX, USA). The mRNA cleavage sites at the 10th position of miRNA alignment with P value < 0.05 were considered as significant. The identified targets were grouped into five categories (category 0-4) as previously described [3,4], based on the abundance of target mRNA tags relative to the overall profile of degradome reads. Category 0 includes degradome tags that with >1 raw read at the position, and the abundance at this position is equal to the maximum on the transcript which only has one maximum value. Category 1 is defined as tags with >1 raw read at the position, with abundance at this position equal to the maximum on the transcript which has over one maximum value. Category 2 is defined as tags with >1 raw read at the position, with abundance at this position less than the maximum but higher than the median for the transcript. Category 3 contains tags with >1 raw read at the position, and abundance at this position equal to or is less than the median for the transcript. Degradome tags in Category 4 show only one raw read at the position.

Supplemental References

1. Liu, H.; Able, A.J.; Able, J.A. Transgenerational effects of water-deficit and heat stress on germination and seedling vigour - new insights from durum wheat microRNAs. *Plants* **2020**, *9*, 189.
2. Li, X.; Shahid, M.Q.; Wu, J.; Wang, L.; Liu, X.; Lu, Y. Comparative small RNA analysis of pollen development in autotetraploid and diploid rice. *Int. J. Mol. Sci.* **2016**, *17*, 499.
3. Sun, W.; Xu, X.H.; Wu, X.; Wang, Y.; Lu, X.; Sun, H.; Xie, X. Genome-wide identification of microRNAs and their targets in wild type and phyB mutant provides a key link between microRNAs and the phyB-mediated light signaling pathway in rice. *Front. Plant Sci.* **2015**, *6*, 372.
4. Ji, Y.; Chen, P.; Chen, J.; Pennerman, K.K.; Liang, X.; Yan, H.; Zhou, S.; Feng, G.; Wang, C.; Yin, G. Combinations of small RNA, RNA, and degradome sequencing uncovers the expression pattern of microRNA-mRNA pairs adapting to drought stress in leaf and root of *Dactylis glomerata* L. *Int. J. Mol. Sci.* **2018**, *19*, 3114.
