# Supplementary figures and images for: Integrated Analysis of Small RNA, Transcriptome, and Degradome Sequencing Reveals the Water-Deficit and Heat Stress Response Network in Durum Wheat
Source: Int J Mol Sci. 2020 Aug 21;21(17):6017. doi: 10.3390/ijms21176017 (PMC7504575; doi:10.3390/ijms21176017)

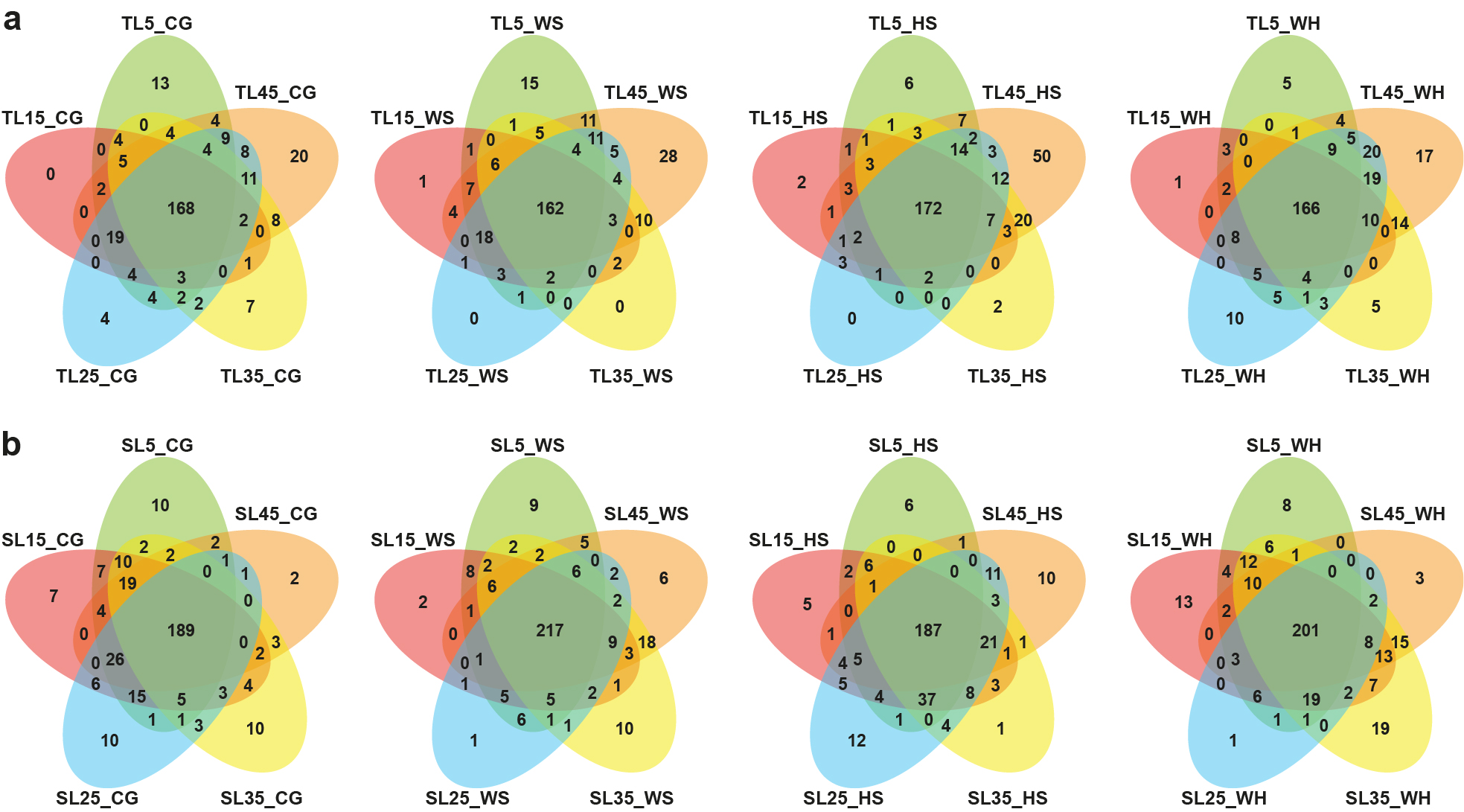

Supplement: Supplementary file 1 [file ijms-21-06017-s001.zip › Supplementary files R2/Figure S1.jpg]

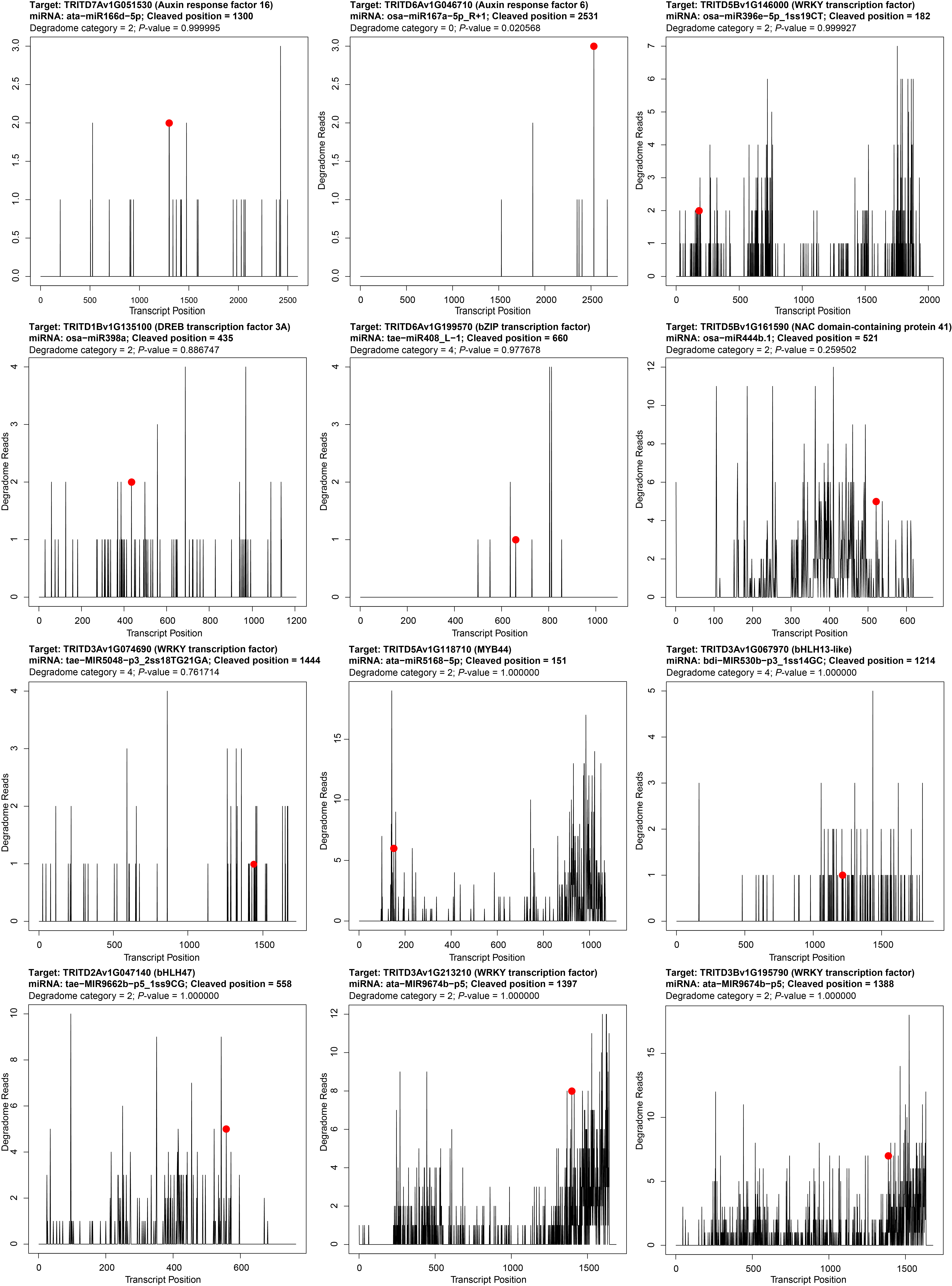

Supplement: Supplementary file 1 [file ijms-21-06017-s001.zip › Supplementary files R2/Figure S10.jpg]

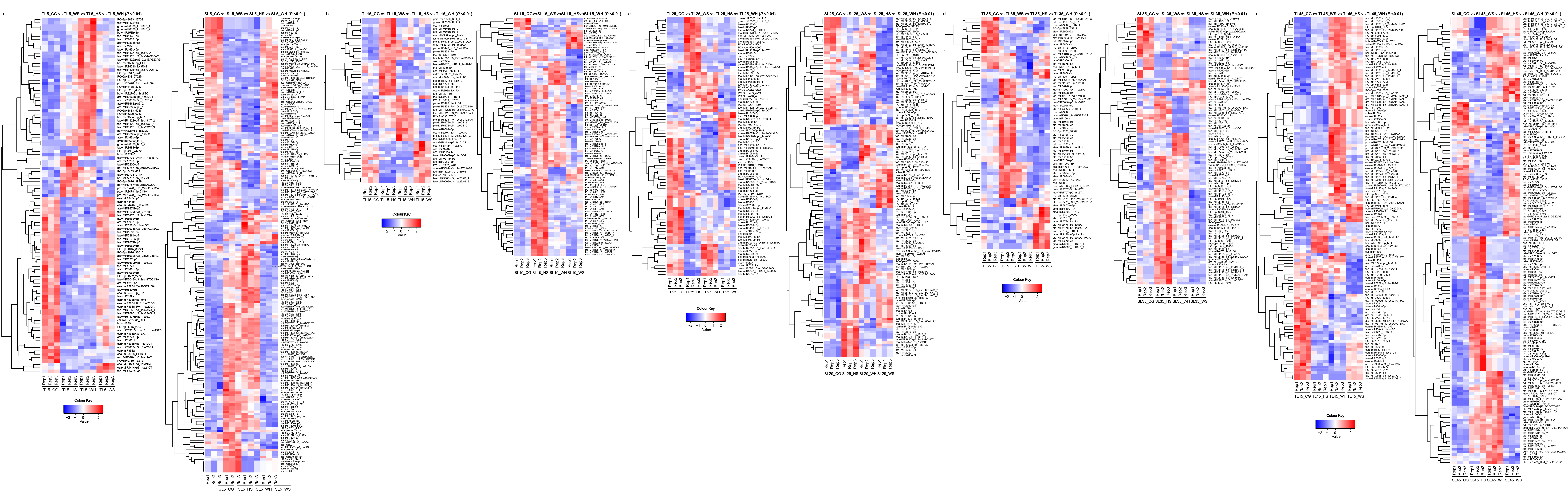

Supplement: Supplementary file 1 [file ijms-21-06017-s001.zip › Supplementary files R2/Figure S2.jpg]

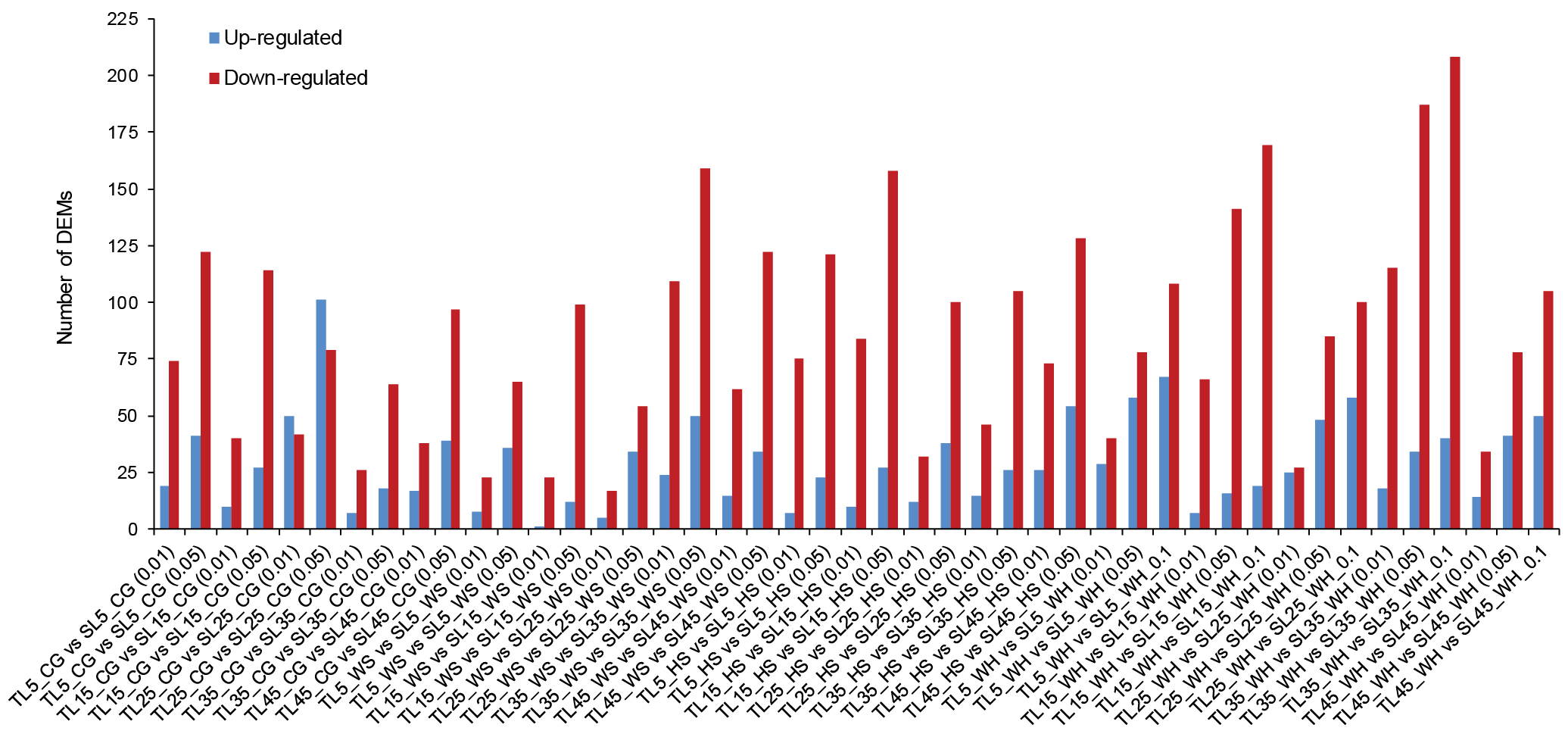

Supplement: Supplementary file 1 [file ijms-21-06017-s001.zip › Supplementary files R2/Figure S3.jpg]

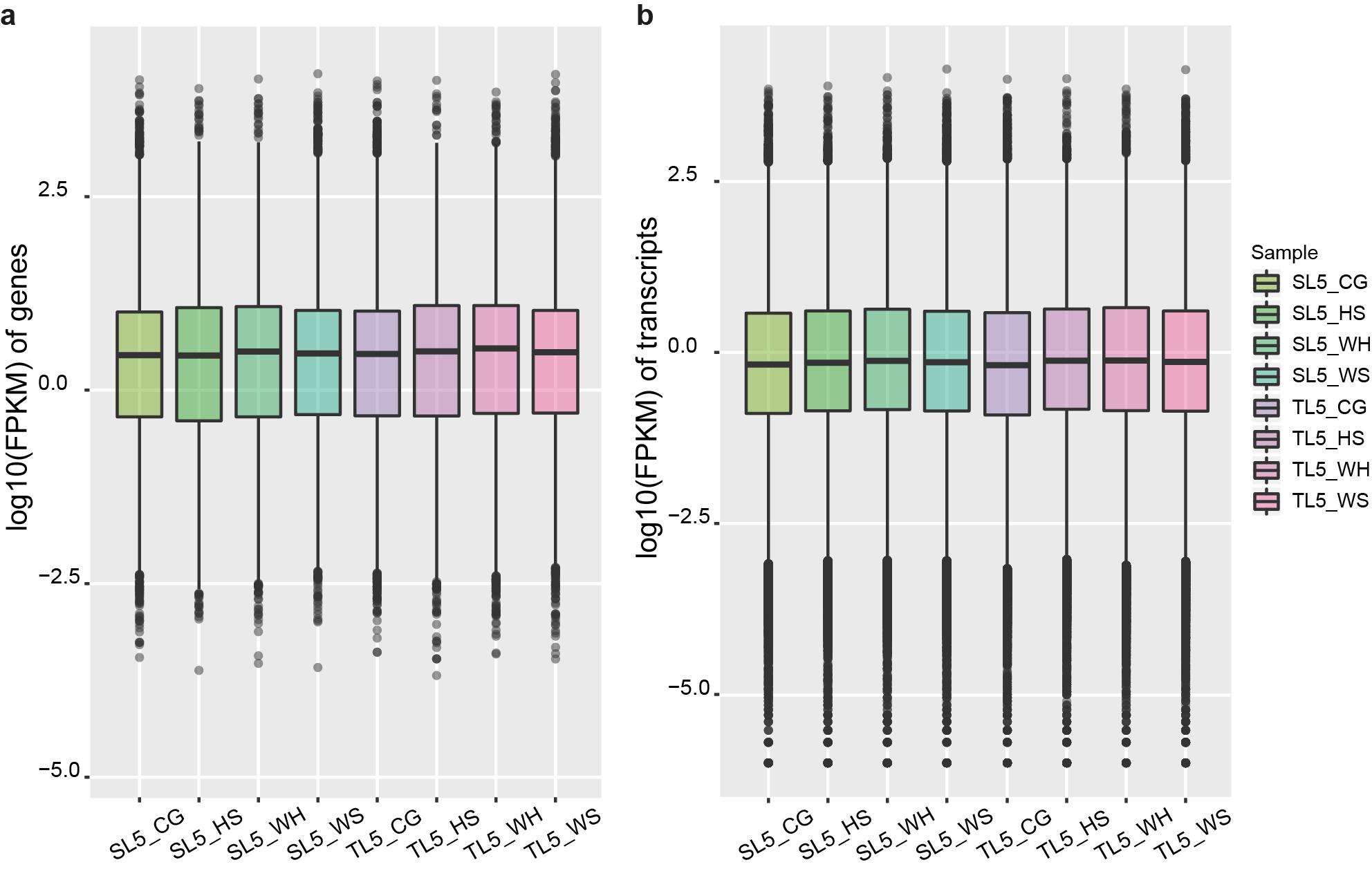

Supplement: Supplementary file 1 [file ijms-21-06017-s001.zip › Supplementary files R2/Figure S4.jpg]

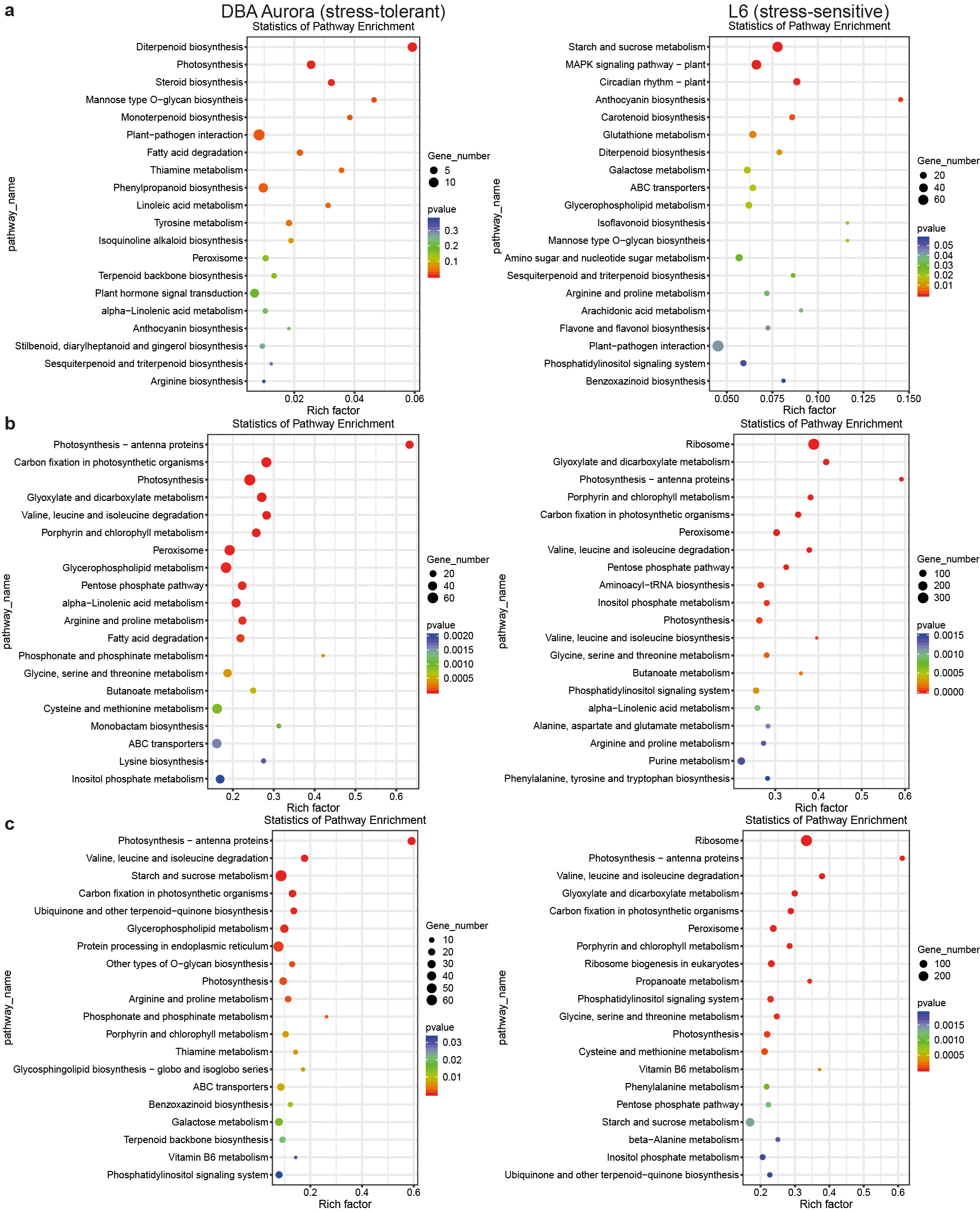

Supplement: Supplementary file 1 [file ijms-21-06017-s001.zip › Supplementary files R2/Figure S5.jpg]

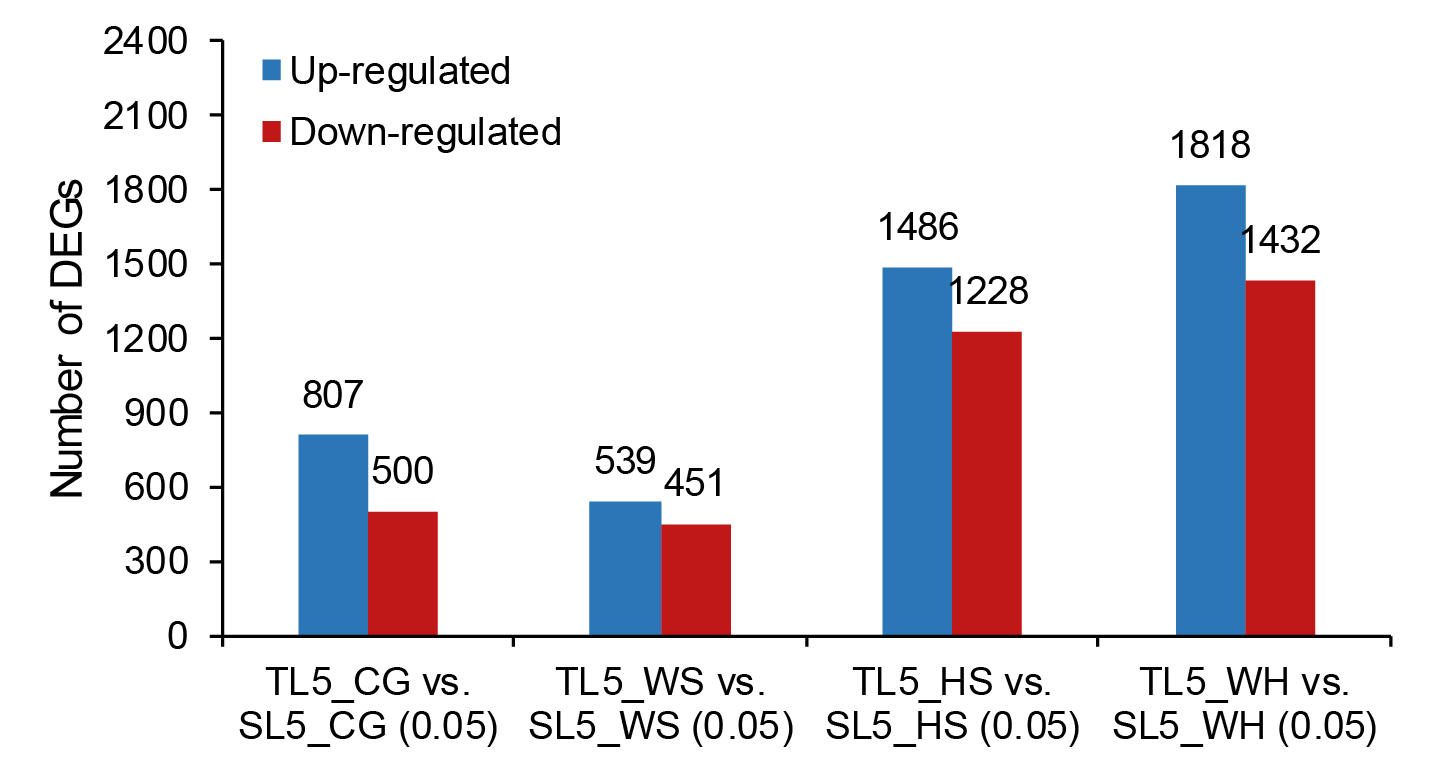

Supplement: Supplementary file 1 [file ijms-21-06017-s001.zip › Supplementary files R2/Figure S6.jpg]

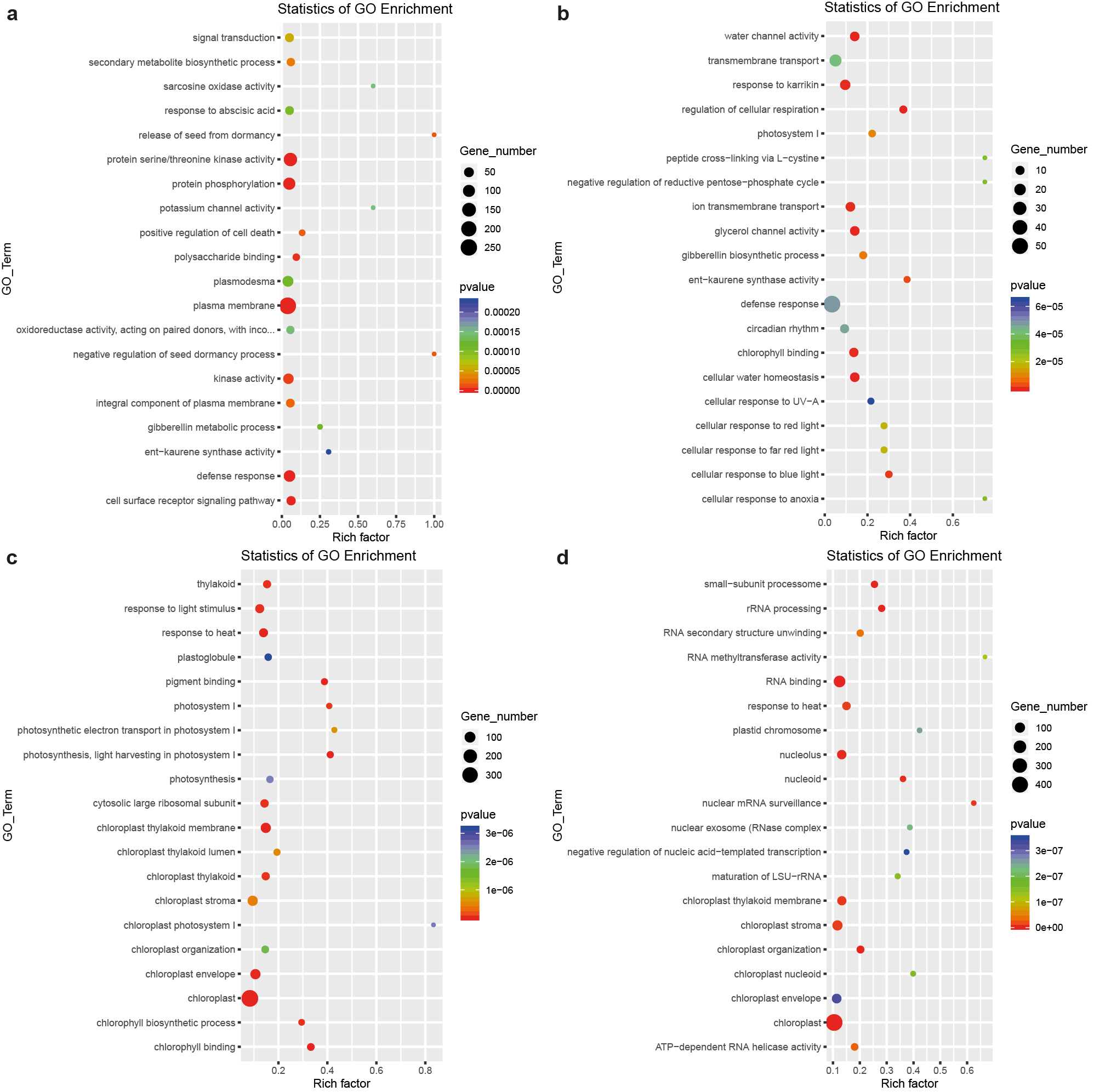

Supplement: Supplementary file 1 [file ijms-21-06017-s001.zip › Supplementary files R2/Figure S7.jpg]

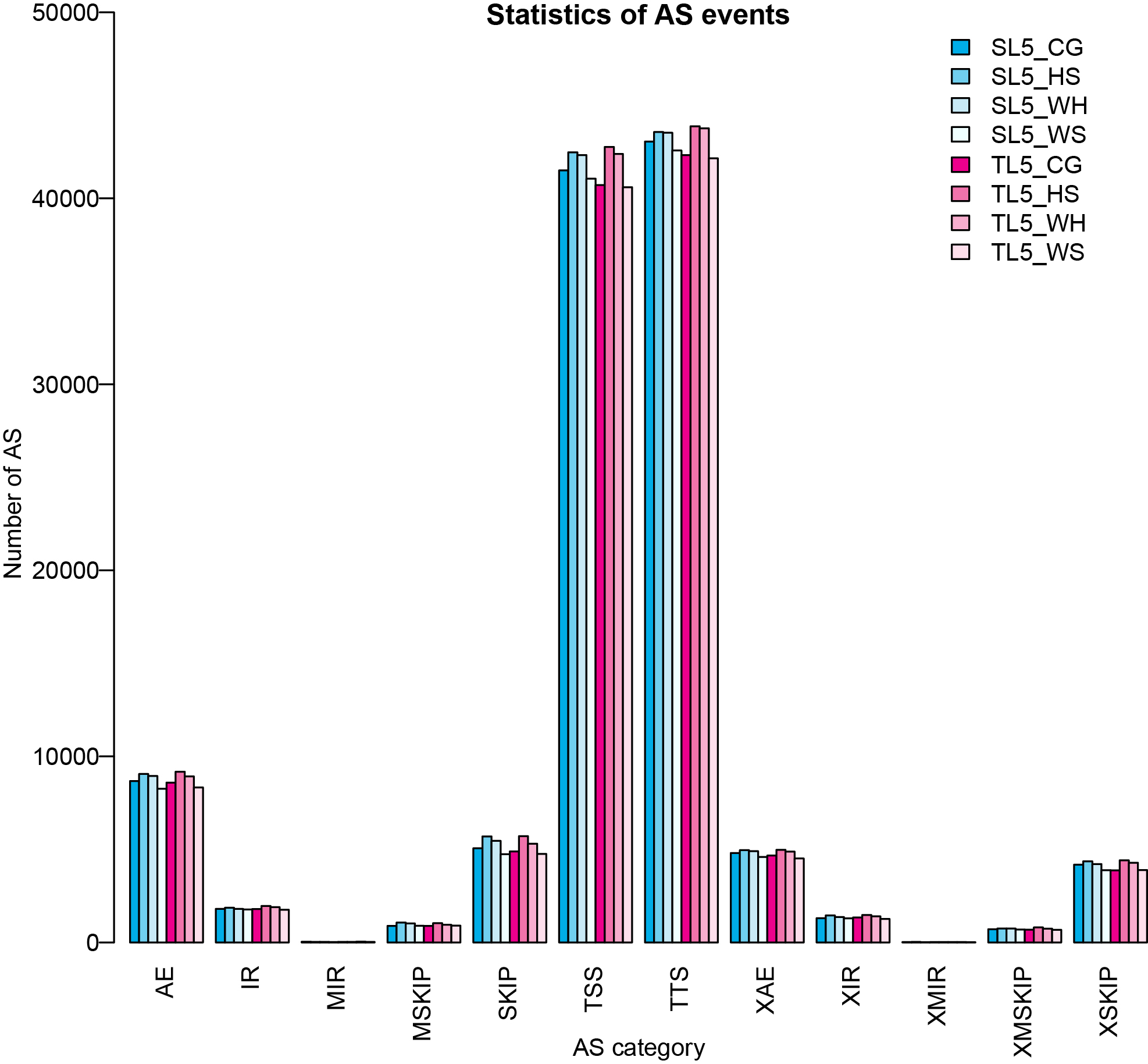

Supplement: Supplementary file 1 [file ijms-21-06017-s001.zip › Supplementary files R2/Figure S8.jpg]

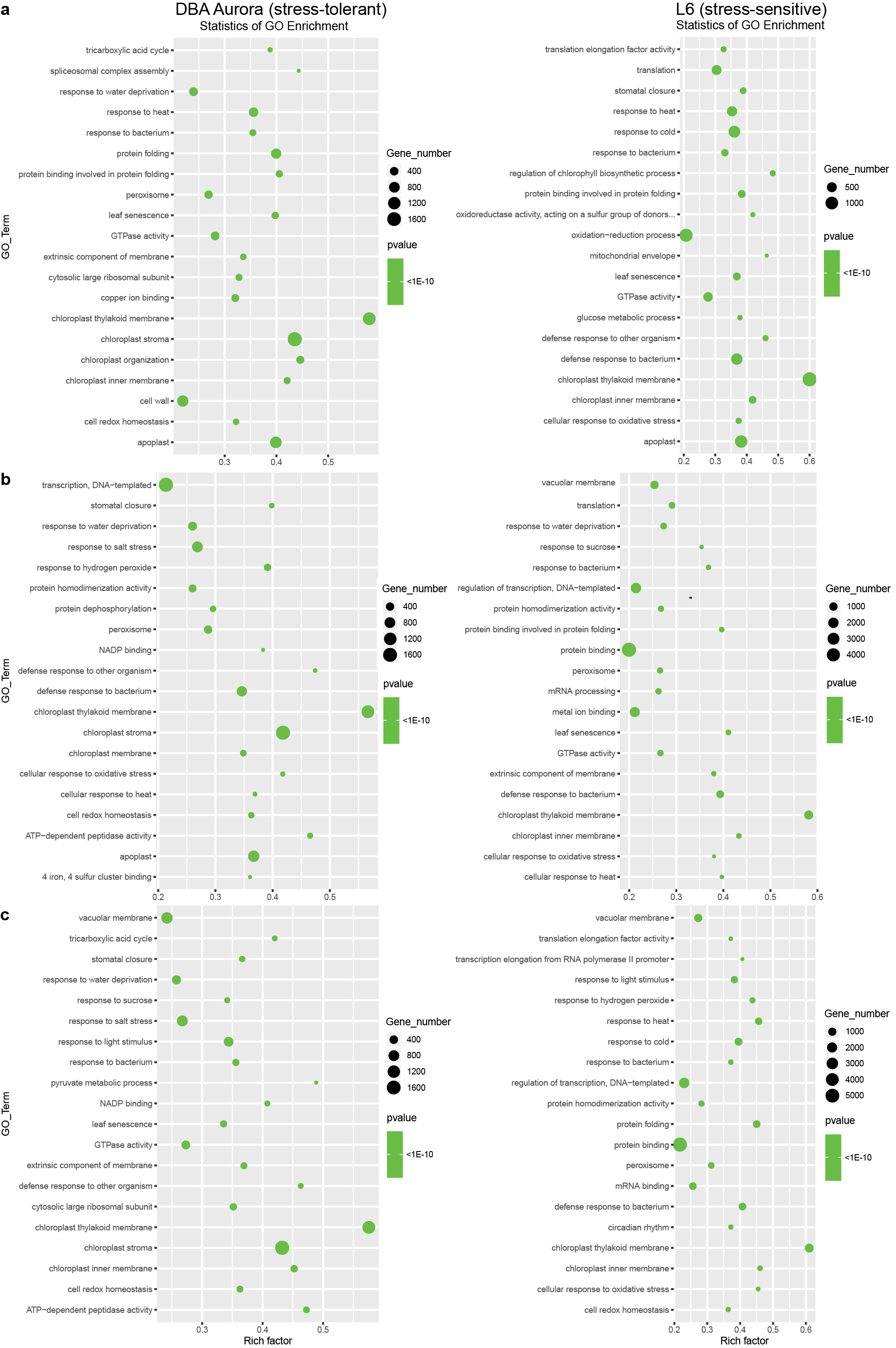

Supplement: Supplementary file 1 [file ijms-21-06017-s001.zip › Supplementary files R2/Figure S9.jpg]
